# Tissue Expression of Carbonic Anhydrase IX Correlates to More Aggressive Phenotype of Basal Cell Carcinoma

**DOI:** 10.3389/fonc.2021.659332

**Published:** 2021-03-25

**Authors:** Daniela Russo, Silvia Varricchio, Gennaro Ilardi, Francesco Martino, Rosa Maria Di Crescenzo, Sara Pignatiello, Massimiliano Scalvenzi, Claudia Costa, Massimo Mascolo, Francesco Merolla, Stefania Staibano

**Affiliations:** ^1^Pathology Unit, Department of Advanced Biomedical Sciences, University of Naples “Federico II”, Naples, Italy; ^2^Dermatology Unit, Department of Clinical Medicine and Surgery, University of Naples “Federico II”, Naples, Italy; ^3^Department of Medicine and Health Sciences “V.Tiberio”, University of Molise, Campobasso, Italy

**Keywords:** basal cell carcinoma, carbonic anhydrase IX, IHC, skin cancer, prognosis, risk stratification

## Abstract

Basal cell carcinoma (BCC) is the most common cancer in the white-skinned population accounting for about 15% of all neoplasms. Its incidence is increasing worldwide, at a rate of about 10% per year. BCC, although infrequently metastasizing, very often causes extensive tissue losses, due to the high propensity toward stromal infiltration, particularly in its dedifferentiated forms, with disfiguring and debilitating results. To date, there still is limited availability of therapeutic treatments alternative to surgery. We evaluated the immunohistochemical expression of the carbonic anhydrase IX (CAIX), one of the main markers of tissue hypoxia, in a set of 85 archived FFPE BCC tissues, including the main subtypes, with different clinical outcomes, to demonstrate a possible relationship between hypoxic phenotype and biological aggressiveness of these neoplasms. Our results showed that the expression level of the CAIX protein contributes to the stratification of BCC in the different risk classes for recurrence. We hypothesize for CAIX a potential therapeutic role as a target therapy in the treatment of more aggressive BCCs, thus providing an alternative to surgical and pharmacological therapy with Hedgehog inhibitors, a promising example of target therapy in BCCs.

## Introduction

Basal cell carcinoma (BCC) is defined by the World Health Organization (WHO) as a locally invasive, slow-growing tumor that originates from the basal layer cells of the epidermis, placed peripherally to the hair bulbs, and that rarely hesitates in metastasis. The main risk associated to BCC are multiple relapses, an event more frequently occurring in case of incomplete excision or multiple primitive tumors, Relapsing BCC can produce, over time, serious anatomic, functional and aesthetic damage, with serious problems of co-morbidity, severely affecting the quality of life (1). Accounting for about 15% of all solid tumors, BCC is the most common malignant neoplasm in the world, with more than 2.8 million new cases diagnosed each year in the United States of America (2). In the context of non-melanoma skin cancer (NMSC), BCC accounts for about 80% of the cases (3), with a global incidence increase of 3% to 7%/year over last decades (4), BCC represents a serious public health problem. In Italy, the incidence is approximately 100 cases per 100,000 inhabitants (5). These figures could be underestimated because of the diagnostic-therapeutic management for this neoplasia. BCC treatment, in fact, does not usually include hospitalization, and BCC generally does not cause patients’ death. BCC develops predominantly in the mature-elderly population (>40 years), prevalently males, with average age at diagnosis of 68 years, in regions of the body chronically exposed to the sun (particularly face and neck, 70% to 85% of cases; 25% to 30% being represented by the nose alone, to follow the trunk and less frequently the limbs). Recently, an epidemiological shift has been reported, with increased incidence in young female population, probably due to the varied habits of exposure of the population (not adequately protected) in the Sun (6). BCC recognizes as the main risk factor exposure to sunlight, especially UVA and UVB ultraviolet rays. Different BCC variants have been described, based on clinical behavior, morphology, growth pattern, architecture, and differentiation (7). Hypoxia is a pathological condition determined by a lack of oxygen in the whole organism (generalized hypoxia) or in one tissue (tissue hypoxia). Hypoxia has emerged as an important feature of tumor microenvironment of neoplasms with more aggressive biological behavior. The uncontrolled growth of tumors is, in fact, accompanied by the induction of insufficient vascularization which results in the formation in most of the malignant solid tumors of heterogeneously distributed hypoxia regions (8, 9). Hypoxia generates a passage to the glycolic metabolism that allows the production of energy in low or absent oxygen conditions and is crucial for the survival of hypoxic cancer cells. Among the molecules most expressed in hypoxia condition are HIF-1 α and carbonic anhydrase IX (CAIX). These molecules are responsible for the process of adapting cells to oxygen deficiency with the formation of new blood vessels, a mechanism that is exploited by tumor tissues to grow and metastasize (10). CAIX belongs to the family of Carbonic Anhydrases (CA), a group of metal zinc-containing enzymes that catalyze the reversible hydration of CO2 in HCO3 and H + ions and has recently emerged as the most promising endogenous marker of cellular hypoxia (10, 11). This reaction is fundamental at the level of cells, tissues, and organs in a wide range of biochemical and physiological processes such as acid-base equilibrium, gas exchange, ionic transport, and carbon dioxide fixation. To date, 15 human isoforms of CA have been characterized that differ in catalytic activity, subcellular localization, and tissue distribution (11). Carbonic Anhydrase IX is encoded by a gene located on chromosome 9 and is a transmembrane isoform with a catalytic site in the extracellular portion and has the highest efficiency for the transport of H + between CAs. It consists of a proteoglycan-like domain at the N-terminal end (involved in adhesion and intercellular communication), an extracellular catalytic domain, a trans-membrane hydrophobic portion and a C-terminal cytoplasmic tail (essential for correct localization on the plasma membrane and proper functioning of the enzyme) (12). It is a tumor-associated protein, as it is expressed in limited quantities in normal tissue, such as the stomach or intestine, and the expression is however limited to the basolateral membrane of epithelial cells endowed with increased proliferative activity, while it is hyper expressed in solid tumor cells linked to a hypoxic phenotype (13). The overexpression of CAIX on the cell membrane of many solid tumors is mediated by the HIF-1 transcription factor and is often associated with poor reactivity to classical radio and chemotherapy. In a recent work, a close association between overexpression of CAIX and the markers of staminality CD44 and Nestin, has been demonstrated in several aggressive and metastasizing neoplasms, with relevance in a series of squamous carcinomas of the tongue (14). This indicates that CAIX action in hypoxic tumors goes beyond intra-tumoral PH control. The clear majority of existing data, in fact, indicates that CAIX has multiple functions in solid tumors, in particular, it plays a key role in encouraging the establishment of chemo-and radio-resistance in the most advanced cases and opens new therapeutic perspectives (14). In the present study, we deepened the role of the Carbonic Anhydrase IX as a possible leading actor and marker of hypoxia in BCC, by evaluating the immunohistochemistry expression of the CAIX protein in a series of archived FFPE BCC tissue samples.

## Materials and Methods

### Patients and Tissue Samples

Formalin-fixed, paraffin-embedded tissue blocks of 85 BCCs, diagnosed and excised with healthy surgical margins from February 2002 to November 2017, were retrieved from the archives of the Pathology Section of the Department of Advanced Biomedical Sciences, “Federico II” University of Naples. Out of 85 cases, 55 males and 30 females, the age at diagnosis ranged between 38 and 88 years (mean age, 67 years). **Table 1** summarizes the histological groups of the study population, together with the associated risk. The clinical data and pathological features of the tumors are reported in **Table 2**. The study design and procedures involving tissue samples collection and handling were performed according to the Declaration of Helsinki, in agreement with the current Italian law, and to the Institutional Ethical Committee guidelines.

**Table 1:** Study population summary grouped by histological types.

Risk of recurrence	Histotype	Count
Higher risk	Basosquamous carcinoma	14
	Infiltrating BCC	34
	Micronodular BCC	4
	Sclerosing/morphoeic BCC	10
Higher risk, total		62
Lower risk	BCC with adnexal differentiation	2
	Nodular BCC	16
	Superficial BCC	5
Lower risk, total		23
Total		85

**Table 2:** Clinical-pathologic characteristics of the study population.

		n	%
Patients	Total	85	100%
Age	Mean	67	
	Range (Min-Max)	38–88	
Sex	Male	55	65%
	Female	30	35%
Tumor site	Area H	46	54%
	Area M	10	12%
	Area L	27	32%
	ND	2	2%
Histologic subtype	BCC with indolent growth	23	27%
	BCC with aggressive growth	62	73%
Follow-up	Recurrence	30	35%
	No recurrence	55	65%
Follow-up time (months)	Mean	39	
	Median	42	
	Min	2	
	Max	153	
Tumor size	>2 cm	25	29%
	<2 cm	59	70%
	N.D.	1	1%

Area H: “mask areas” of face (central face, eyelids, eyebrows, periorbital, nose, lips [cutaneous and vermilion], chin, mandible, preauricular, and postauricular skin/sulci, temple, ear), genitalia, hands, and feet; Area M: cheeks, forehead, scalp, neck, and pretibial; Area L: trunk and extremities; BCC with indolent or ordinary growth: BCC with solid nest, superficial, adenoid, keratotic; BCC with aggressive or aggressive growth: BCC morphoeic, basosquamous, micronodular, dedifferentiated.

### TMAs Construction and Immunohistochemistry

Two pathologists (SS and DR) reviewed the whole routine hematoxylin-eosin (H&E) sections to confirm the original diagnosis and to mark the most representative tumor areas useful for the TMA construction. Tissue cores with a diameter of 3 mm were punched from morphologically representative tissue areas of each “donor” tissue block and brought into one recipient paraffin block using a manual tissue arrayer. The filled recipient blocks were then placed on a metal base mold. The paraffin-embedding was then carried-out, by heating the blocks at 42°C, for 10 min, and flattening their surface by pressing a clean glass slide on them. As a result, four TMAs were built. 4-μm sections were cut from each TMA using an ordinary microtome (15, 16). The first section was stained with H&E to confirm the presence of the tumor and the integrity of tissues. The other section was mounted on a super frost slide (Microm, Walldorf, Germany) for the immunohistochemical evaluation of CAIX. For CAIX IHC assay the sections were deparaffinized routinely in xylene and rehydrated through a series of graded ethanol. CAIX antigen retrieval was performed in EDTA buffer (pH 8) in a hot water bath (94°C) for 20 min and in CITRATE buffer (pH 6) by microwave oven (3 min × 3 times); the backdrop (for blocking non-specific background staining) was removed using the universal blocking serum (Dako Diagnostics, Glostrup, Denmark) for 15 min at room temperature. Endogenous alkaline phosphatase activity was quenched adding Levamisole to buffer AP (Substrate Buffer); the slides were rinsed with TRIS+Tween20 pH 7.4 buffer and incubated in a humidified chamber with the primary rabbit polyclonal antibody anti-CAIX (sc-25599, Santa Cruz Biotechnology, diluted 1:200 overnight at 4°C). Then used a biotinylated secondary antibody and streptavidin conjugated with alkaline phosphatase. The reaction has been highlighted with the chromogen Fast Red, which showed the presence of antigen that we sought in red (Dako REAL Detection System, Alkaline Phosphatase/RED, Rabbit/Mouse). Again, after a weak nuclear counterstain with hematoxylin, the sections were then mounted with a synthetic medium (Entellan, Merck, Darmstadt, Germany). Positivity for CAIX was visualized as red membranous and cytoplasmic staining. The CAIX expression was defined as high or low depending on whether the percentage of neoplastic cells stained was respectively >/= or <5%.

### Statistical Analysis

Correlation between CAIX immunohistochemical expression and BCC clinical-pathologic characteristics was asses through contingency analysis with Fisher exact test. Statistical analysis has been performed using SPSS software (IBM Corp. Released 2013. IBM SPSS Statistics for Windows, Version 22.0. Armonk, NY: IBM Corp.).

## Results

**Figure 1:**
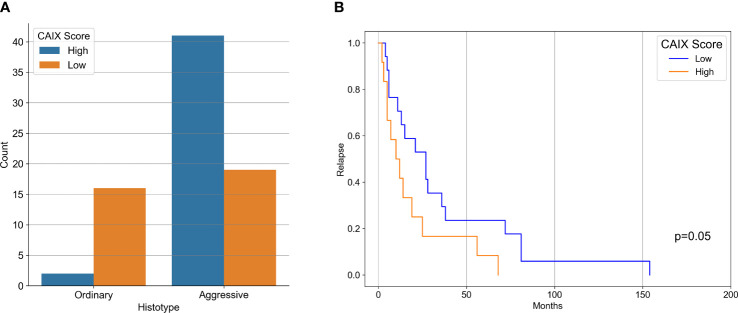
**(A)** Bar-Graph representation of CAIX immunohistochemical expression in aggressive and ordinary BCC. **(B)** Kaplan-Meier curves of recurrence survival. Difference between CAIX HIGH and CAIX LOW curved proved statistically significant (p = 0.05) as tested by Log-Rank.

Our case series included 85 tumor samples (**Table 2**), out of which, 7 (8%) were not evaluable for CAIX tissue expression due to loss of core integrity. The CAIX protein showed LOW expression score in 35 (45%) out of 78 cases, and a HIGH score in the residual 43 (55%) (**Table 3**). The study population was subdivided, according to the histotype, into two groups: aggressive BCCs (i.e., BCCs with higher risk of recurrence; including basosquamous, morphoeic, infiltrating and micronodular) consisting of 60 cases (60/78, 77%) and the group of ordinary BCCs (i.e., BCCs with lower risk of recurrence; including nodular, superficial, and with adnexal differentiation) consisting of 18 cases (18/78, 23%) (**Table 1**). Among aggressive BCC, 41 out of 60 evaluable cases (68.3%) showed a HIGH CAIX expression score, while in the group of ordinary BCCs only 2 out of 18 cases (11.1%) showed a high score (**Table 4**; **Figure 1A**). **Table 5** shows the distribution of CAIX expression scores per histologic subtypes. The follow-up data for recurrence, detailed per tumor variants, are shown in **Table 6**.

**Table 3:** CAIX IHC tissue expression score frequency distribution in the studied population.

CAIX expression score frequency distribution				
CAIX		Frequency	Percentage (Total)	Percentage (Valid)
Valid	Low	35	41%	45%
	High	43	51%	55%
	Tot. Valid	78	92%	100%
Missing		7	8%	
Total		85	100%	

**Table 4:** Contingency table of BCC histologic classification by CAIX score.

Contingency Table Classification * CAIX score				
		CAIX score		
		High	Low	Total
Classification	Aggressive	41 (68.3%)	19 (31.7%)	60 (100%)
	Ordinary	2 (11.1%)	16(88.9%)	18 (100%)
Total	43(55.1%)	35 (44.9%)	78 (100%)	

**Table 5:** Crosstab of CAIX expression by histologic subtypes.

Histologic type	CAIX score		
	High	Low	Total
Basosquamous carcinoma	9 (64.3%)	5 (35.7%)	14 (100%)
BCC with adnexal differentiation	0	2 (100%)	2(100%)
Infiltrating BCC	22 (66.7%)	11 (33.3%)	33 (100%)
Micronodular BCC	2 (50%)	2 (50%)	4 (100%)
Nodular BCC	1 (8.3%)	11 (91.7%)	12 (100%)
Sclerosing/morphoeic BCC	8 (88.9%)	1 (11.1%)	9 (100%)
Superficial BCC	1 (25%)	3 (75%)	4 (100)

**Table 6:** Crosstab of recurrence follow-up data by tumor variants.

Histologic type	Follow-Up		
	Not recurrent	Recurrent	Total
Basosquamous carcinoma	10	4	14
BCC with adnexal differentiation	1	1	2
Infiltrating BCC	15	18	33
Micronodular BCC	1	3	4
Nodular BCC	11	1	12
Sclerosing/morphoeic BCC	5	4	9
Superficial BCC	3	1	4
Total	46	32	78

**Figure 2:**
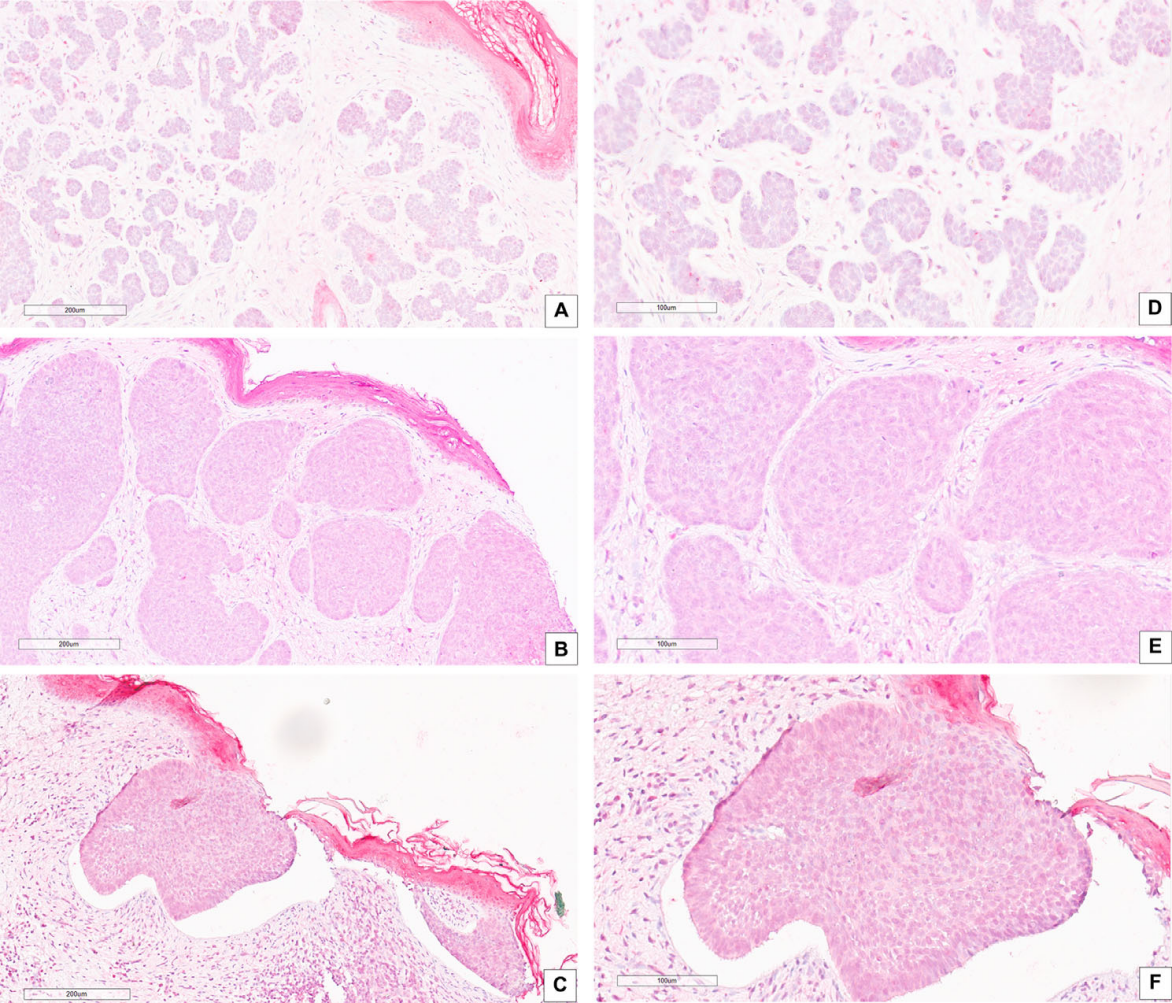
IHC stain with an anti-CAIX antibody in low risk BCC histological variants: **(A–D)** BCC with adnexal differentiation (magnification, 20× and 40×, respectively); **(B–E)** Nodular BCC (magnification, 20× and 40×, respectively); **(C–F)** Superficial BCC (magnification, 20× and 40×, respectively). Scale bars are shown.

**Figure 3:**
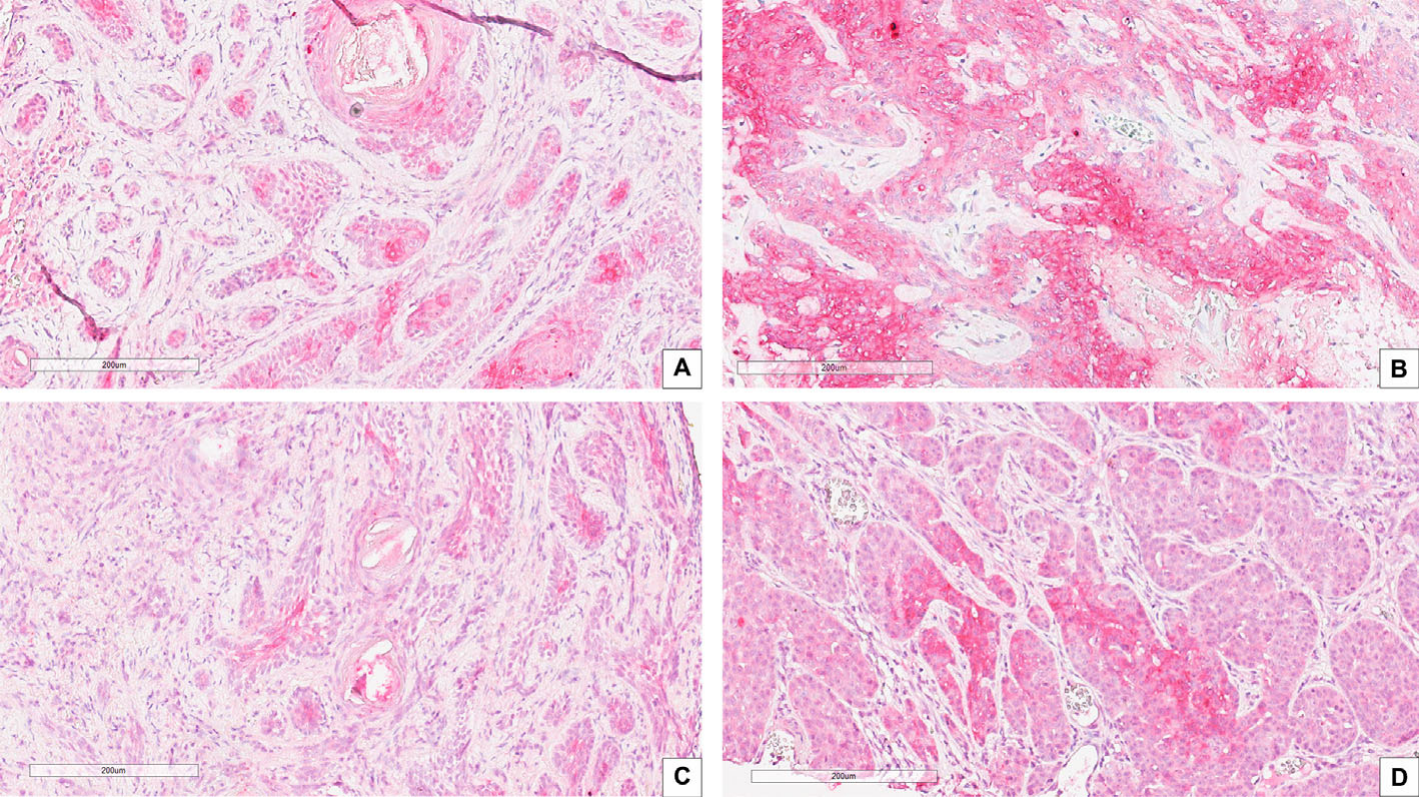
IHC stain with an anti-CAIX antibody in high risk BCC histological variants: **(A)** Infiltrating BCC; **(B)** Morpheaform BCC; **(C)** Micronodular BCC; **(D)** Basosquamous BCC. Scale bars are shown, magnification is 20×.

Correlation between CAIX immunohistochemical expression and BCC histotype was assessed through contingency analysis with Fisher exact test, that proved to be statistical significant, with a P value <0.0001. A survival analysis, taking recurrence as endpoint, was carried out and Kaplan-Meier curves are shown in **Figure 1B**: difference between CAIX HIGH and LOW curves is significant as resulted from Log-Rank test (p = 0.05). Taken together our results show that the higher CAIX expression significantly correlates with BCC aggressive behavior. Representative images of CAIX IHC staining in low-risk BCCs are shown in **Figure 2**; representative high-risk BCCs immunostained with anti-CAIX antibody are shown in **Figure 3**.

## Discussion

Basal carcinoma (BCC) represents 15% of all neoplasms and constitutes a serious public health problem, being the most common cancer in the white-skinned population. Its incidence is increasing worldwide, with an increase of ≥ 10%/year [Lomas et al. (2)]. BCC is a tumor that, despite its low frequency of distant metastasis, frequently causes extensive tissue losses, due to a marked tendency to stromal infiltration, particularly in its dedifferentiated forms, with disfiguring and debilitating results. The maximum expression of this aggressive behavior is the so-called Ulcus Rodens with destructive consequences for the cartilage and bone tissues. To date, there is still limited availability of alternative therapeutic treatments to surgery. Recently, promising results seem to emerge from the early follow-up of patients treated with molecular anti-Sonic Hedgehog therapy, a pathway associated with the BCC carcinogenesis process. It is compelling to unravel the pathogenetic mechanisms underlying the aggressiveness potential of each BCC subtypes, in order to achieve an effective personalized therapy for these tumors. The need of greater understanding of BCC biology appears even more urgent given that this neoplasia preferentially affects the adult and elderly population and that prevention and early diagnosis are still unattained goals, especially in emerging areas of the World and in Western countries peripheral areas. In recent years, a large body of data has highlighted the importance of the interaction of cancer cells with the tumor microenvironment, which provides support for the growth and development of neoplasia. This assumption brought to light new study hypotheses, in order to characterize the heterotypic interaction between the tumor and its microenvironment. Among the alterations of the tumor microenvironment, much attention has been paid, in recent decades, to hypoxia, a pathophysiological feature of locally advanced tumors, resulting from genetic instability, diminished apoptotic potential, and angiogenesis. The hypoxic state also plays an important role in relapsing, metastasis and poor response to treatments, including radiotherapy, chemotherapy and angiogenic treatment. Among the molecules most expressed in hypoxic condition, the carbonic anhydrase IX (CAIX) is considered a marker of hypoxia *in vivo* (17), whose overexpression has been correlated with increased tumor aggression in different types of cancer (18–22). To date, a series of CAIX inhibitors have been synthesized, both in the form of small inhibitory molecules and as monoclonal antibodies, used as antitumor agents in different models of neoplasms (23–31). In a previous work we tested the expression of CAIX in several human solid tumors, extending the CAIX expression information to the expression of the stem cells markers CD44 and nestin in solid cancers, to explore their relationship with the biological behavior of tumors. We found that CAIX is strongly expressed in advanced tumors, including squamous cell invasive cancer of the tongue (14). The role of CAIX as a prognostic biomarker in oral cancer has been recently reviewed by (32), whose systematic review and meta-analysis showed that that immunohistochemical CAIX assessment is a useful OSCC prognostic biomarker. In the present work, the immunohistochemistry expression of the CAIX protein was evaluated in a selected series of patients with BCC divided into two groups based on the histological subtype. The highest levels of protein were found in the aggressive BCC group consisting mainly of morphoeic BCC and Basosquamous BCC. 68.3% of cases showed a high level of expression, and the remaining 31.7% a low level. CAIX expression frequency distribution has been reported in all the histotypes described in our case series, and the statistics of CAIX expression correlation with BCC subtypes have been carried out grouping BCC samples into two categories, aggressive and ordinary one, according to clinical behavior of each subtype, in order to overcome the relative small number of subjects for some subtypes. In the ordinary group, 92% of cases expressed low levels of CAIX and only 8% show a high score. The Fisher Exact Test confirmed that the difference in CAIX immunostain observed between the two BCC groups was statistically significant. The most significant result was obtained by comparing the averages of expression between the aggressive and ordinary groups, and the difference showed a value of P<0.0001. In conclusion, these results suggest that the expression levels of the CAIX protein can help to stratify BCCs in different risk classes; moreover, our results let envisage a role for CAIX as a therapeutic target to counteract the most aggressive BCC, providing a viable alternative to the surgical approach, and to the inhibitors of Hedgehog Pathway, a promising tool for target therapy in BCCs, often associated with various degrees of toxicity (muscle spasms, alopecia, dysgeusia, weight loss, fatigue, nausea, decreased appetite and diarrhea) that in the most severe forms (hypovolemic shock, myocardial infarction, meningeal disease, Ischemic stroke) determine the interruption of treatment, with no resolution of the pathology in place.

## Data Availability Statement

The raw data supporting the conclusions of this article will be made available by the authors, without undue reservation.

## Ethics Statement

The study was performed according to the Italian law, and according to the Declaration of Helsinki for studies based only on retrospective analyses on routine archival FFPE-tissue.

## Author Contributions

All authors listed have made a substantial, direct and intellectual contribution to the work, and approved it for publication. All authors contributed to the article and approved the submitted version.

## Funding

The Pathology Section of the University of Naples “Federico II”, Department of Advanced Biomedical Sciences, was funded by a POR Campania FESR 2014-2020 grant; “Technological Platform: eMORFORAD-Campania” grant PG/2017/0623667.

## Conflict of Interest

The authors declare that the research was conducted in the absence of any commercial or financial relationships that could be construed as a potential conflict of interest.
